# A comparison of applicant and accepted student characteristics to research training programs with implications for recruitment and selection strategy

**DOI:** 10.3389/feduc.2025.1474591

**Published:** 2025-02-05

**Authors:** Young-Hee Cho, Chi-Ah Chun, Hector Ramos, Paul Buonora, Vasanthy Narayanaswami, Kim-Phuong L. Vu

**Affiliations:** 1 Department of Psychology, California State University, Long Beach, CA, United States; 2 CSULB BUILD Program, California State University, Long Beach, CA, United States; 3 Department of Chemistry and Biochemistry, California State University, Long Beach, CA, United States; 4 CSULB RISE Program, California State University, Long Beach, CA, United States; 5 CSULB MARC U*STAR Program, California State University, Long Beach, CA, United States

**Keywords:** demographic characteristics, psychosocial characteristics, application, acceptance, undergraduate research training, diversity

## Abstract

**Introduction:**

Very few studies have examined the relationship between student characteristics and their acceptance to research training programs that use holistic selection. The present study addressed this question using institutional and applicant data of three NIH undergraduate training programs at California State University, Long Beach. Its first aim was to examine whether the applicants to the training programs were representative of the broader campus population. Its second aim was to investigate whether applicants who were accepted to the programs using a holistic selection process differed in academic discipline, demographics, and psychosocial characteristics from applicants who were not accepted.

**Methods:**

Information on students’ majors, race/ethnicity, and gender was obtained from the university records or applications submitted by students. Majors were categorized as either biomedical or behavioral disciplines, while URM status was defined as students who self-identified their race and ethnicity as African American/Black, Native American, Native Hawaiian/Pacific Islander, or Hispanic. Applicants’ psychosocial characteristics were obtained from a separate online survey. The acceptance status of applicants was obtained from the training programs’ records.

**Results:**

The applicant and non-applicant groups showed similar distribution of demographic characteristics regarding URM status and gender. Moreover, students’ academic discipline and other demographic variables were not associated with application status at either the lower division (LD) or upper division (UD) levels. Although psychosocial characteristics measured with the online survey were not considered in the selection process, post-hoc analyses showed that LD applicants with higher grit and UD applicants with higher science interests were more likely to be accepted to the programs.

**Conclusion:**

The equal representation of URM and female students in the applicant and non-applicant groups suggests that students from these traditionally underrepresented groups in STEM were just as likely to apply to our training programs. Furthermore, while the holistic selection process resulted in comparable acceptance rates across URM status and gender, it appeared to favor LD applicants with higher grit and UD students with higher science interests. These findings imply that research training programs can effectively recruit diverse students from underrepresented populations in STEM by using intentional outreach and recruitment efforts coupled with an objective and holistic selection process.

## 1 Introduction

Recent trends in higher education call for increased spending and focus on developing and producing greater numbers of higher numbers of graduates in Science, Technology, Engineering and Math (STEM) fields. While there has been an increase in high school graduation of underrepresented minority students who enter college and an increase in those students who complete their degree in the field of science and pursue advanced degrees, their numbers are still behind those of well-represented students (Hurtado et al., 2008; National Science Foundation, 2024). The smaller number of URM students in the field of science results in fewer researchers who are more likely to address the issues of under-represented populations in health-related fields (Hurtado et al., 2008). Fortunately, research on undergraduates in STEM shows that substantial involvement in faculty-led research, particularly at an early stage of undergraduate studies, improves students’ retention and graduation rates and pursuit of careers in science, including for students from groups historically underrepresented in STEM (Gilmore et al., 2015; Hernandez et al., 2018; Hurtado et al., 2008). Additionally, intensive undergraduate research training has been found to be effective both at the lower and upper division levels in increasing students’ research knowledge and skills, science identity, and matriculation in graduate programs (Eagan et al., 2013; Hall et al., 2016; Hurtado et al., 2017; Kingsford et al., 2022; Vu et al., 2023). More importantly, students from historically underrepresented groups in STEM appear to benefit from such training programs as much as those from well-represented groups (e.g., Estrada et al., 2016; Hall et al., 2016; Vu et al., 2023). However, little is known about who applies to these undergraduate research training programs. Underrepresentation can occur at different points along the pipeline. To fill this gap, the present study examined the rates of application and acceptance to federally funded undergraduate research programs at California State University, Long Beach (CSULB), a Hispanic-Serving Institution (HSI) and Asian American Native American Pacific Islander-Serving Institution (AANAPISI), across several relevant academic disciplines, demographic, and psychosocial characteristics. Such information can help shed light on what might contribute to the continued underrepresentation of students from historically disadvantaged groups.

In response to a critical need to diversify the nation’s biomedical research workforce and eliminate health disparities, the National Institutes of Health (NIH) has, for decades, funded training programs that support the development of historically underrepresented students (e.g., African American, Latine, Native American, Pacific Islander, and women) pursuing research careers in health-related disciplines. The charge for programs such as the Minority Biomedical Research Support (MBRS) and Minority Access to Research Careers (MARC) was to “provide research training opportunities to students and faculty from minority groups underrepresented in the biomedical and behavioral sciences relevant to biomedicine” (e.g., NIH PAR 99–091, 1999). However, the climate for such minority research training programs was forced to change beginning in the mid-1990s with the passing of California Proposition 209 in 1996 that banned affirmative action policies in the state of California and the U.S. fourth Circuit Court of Appeals decision in the 1994 case of Podberesky v. Kirwan that ruled against the University of Maryland’s scholarship program that was exclusively for African American students. When race and gender-based admission became illegal, programs that limited the eligibility to only historically underrepresented groups had to open their programs to non-minority students (Mervis, 2003). Eventually, in 2016, the NIH changed the name of MARC from “Minority Access to Research Careers” to “Maximizing Access to Research Careers” to emphasize its broadened mission to diversify the biomedical research workforce that goes beyond the four historically underrepresented minority ethnic groups.

While targeted outreach and recruitment for students from underrepresented groups is still legal and required by these federally funded programs, the impact of race/ethnicity and gender-neutral selection on diversity of applicant and trainee pools was feared to be devastating (Mervis, 2003). To ensure the trainee pool is diverse, training programs began to adopt a holistic review process of applications that goes beyond traditional measures of academic achievement and potential, such as GPA and standardized test scores. Non-academic factors such as an applicant’s life experiences that demonstrate a genuine interest and motivation in a biomedical research career and bring diverse perspectives to research (Sedlacek, 2005) began to carry increased weight in evaluating applications and selection interviews. While there is no consensus yet on how to conduct a holistic review, early research on holistic admission processes shows promising results, linking it to greater diversity in the admission pools of graduate and professional degree programs (e.g., Henderson et al., 2023; Lewis et al., 2021) than using the traditional metrics. However, we are unaware of any research on the impact of holistic selection processes on the characteristics of accepted applicants to undergraduate research training programs.

The importance of psychosocial characteristics has been reported in higher education literature (Dweck, 2006; Eagan et al., 2023; Miller and Orsillo, 2020; Radunzel et al., 2016; Ramos et al., 2024). In the context of admission, these non-academic traits serve as important indicators of psychological resilience that can foster (and thus predict) success for students from historically underrepresented groups in the graduate and research pipeline in STEM. *Grit* defined as the combination of passion and perseverance for long-term goals (Duckworth and Quinn, 2009) has been linked with school motivation, academic conscientiousness, and academic performance (Christopoulou et al., 2018). Studies on *growth mindset* found it to be beneficial for developing resilience when faced with challenging school transitions and challenging math courses (Yeager and Dweck, 2012). *Science interest* has been linked with persistence and completion of a STEM degree (Radunzel et al., 2016) while general and researcher *self-efficacy* predicted many research related concepts, including intention to pursue research, research identity, and research productivity (Chemers et al., 2011; Frantz et al., 2017; Livinƫi et al., 2021). Students majoring in health and natural sciences also appeared to be more motivated by *intrinsic work values* than those in other majors (Balsamo et al., 2013; Johnson and Elder, 2002).

In sum, the present study attempts to fill the gap in the literature by studying the characteristics of students who applied to the NIH-funded undergraduate research training programs at CSULB. We sought to answer the following three broad questions:

1. Are the applicants representative of our diverse student population on academic discipline and demographic variables associated with historical underrepresentation in STEM?
2. Are the students accepted into the training programs representative of the applicant pool on academic discipline and demographic variables associated with historical underrepresentation in STEM?
3. What psychosocial characteristics of applicants are being captured by our holistic selection process that are predictive of acceptance to the training programs?

## 2 Methods

### 2.1 Training programs

The three NIH-funded undergraduate research training programs were the BUilding Infrastructure Leading to Diversity (BUILD), Maximizing Access to Research Careers Undergraduate Student Training in Academic Research (MARC U*STAR), and Research Initiative for Student Enhancement (RISE) Programs. While the three programs shared the mission of the NIH to enhance the diversity of our nation’s health-related research workforce, the targeted disciplines and student populations varied somewhat. The BUILD Program was a scaled-up program with the largest number of trainees (averaging around 130 per year) and offered training to students from both biomedical and behavioral sciences at both lower-division (LD) and upper-division (UD) levels. The LD program was designed as an early research intervention targeting sophomore-level students who are interested in exploring research. The UD program targeted juniors and seniors who were interested in obtaining intensive research training designed to make them more prepared and competitive for entry into Ph.D. programs in health-related disciplines. The MARC U*STAR Program (10 trainees per year) aimed to develop a diverse pool of honors undergraduates who want to pursue a Ph.D. or M.D./Ph.D. in biomedical and behavioral fields. MARC U*STAR only offered UD training. The RISE Program (20–25 students per year) trained students also in biomedical and behavioral fields and at LD and UD levels. While all three programs accepted trainees in behavioral fields, BUILD had a broader definition of eligible behavioral disciplines as well as the largest numbers of behavioral trainees. It also engaged in more intentional outreach and recruitment from the behavioral sciences. These three training programs were implemented separately with their own grant funding and research training curriculum. However, because of the shared overall mission, a joint application was developed and implemented to minimize the burden on students to apply to multiple programs, allow the program directors to identify which program would be most suitable for the accepted students, and enhance administrative efficiency.

### 2.2 Participants

Participants consisted of two groups: The first group consisted of undergraduate students from the representative majors (see Appendix A) of our training programs (*N* = 28,020) who first enrolled in the university during the fall semester of 8 recruiting cycles of 2015–2023. The second group consisted of applicants to the three NIH training programs (*N* = 246 for LD and 805 for UD). LD applicants were sophomore-level students with at least 3 years remaining until graduation who applied to the two Associate Programs (BUILD and RISE) designed to introduce students to research and research careers. Applicants to the LD programs had little or no prior research experience and were interested in learning about research and research careers. UD applicants were juniors and seniors who applied to any or all of the three NIH UD training programs (BUILD, MARC U*STAR, and RISE) that provide intensive faculty-mentored research training and professional development support. Applicants to the UD programs tended to have some prior research experience and a solid interest in pursuing graduate school in a biomedical or behavioral science discipline. Detailed descriptions of the LD and UD BUILD program components are available in Kingsford et al. (2022) and Vu et al. (2023), respectively. More information about the demographic characteristics of the participants can be found in section 2.5: Students’ academic and demographic information (see Supplementary Tables 1–4). The three training programs were continuously funded by the NIH during the data collection phase between 2015 and 2023. The LD applicants were pooled from 4 recruiting cycles (2015–2019) because the BUILD Associate program was discontinued in the Summer of 2019. The UD applicants were pooled from 8 recruiting cycles (2015–2023).

### 2.3 Outreach and recruitment

The on- and off-campus outreach and recruitment were conducted jointly by the three NIH-funded campus programs. The goals of outreach and recruitment were to raise students’ awareness of undergraduate research opportunities and resources on campus and increase their understanding of the benefits of engaging in research with faculty. The joint efforts were also designed to reduce undergraduates’ confusion about the goals of the three NIH-funded programs and increase administrative efficiency. Ultimately, we wanted to reduce barriers for students by streamlining the application process. To achieve NIH’s goal of increasing the number of URM students pursuing a doctoral degree and research careers in health-related disciplines, we employed intentional strategies to distribute resources to reach students, particularly URM students, from the targeted disciplines. On-campus outreach and recruitment included posting flyers and marketing materials across campus and on the BUILD Program website,^1^ hosting multiple in-person and virtual information sessions, and making presentations at classes and student organization meetings, hosting information tables at campus events, and creating and maintaining a campus-wide searchable online faculty research mentor directory that lists mentors from various training programs.

In recognition of the potential barriers, such as mistrust of science, academia, and faculty, intentional efforts were made to reach out to student service centers and student organizations that serve students from minoritized communities. Moreover, the science/engineering curriculum was enhanced to infuse research into lower and upper-division courses and offer new courses on interdisciplinary research on health disparities, scientific research communications, and research methods courses (Arruda et al., 2024; Taing et al., 2022). These multi-pronged approaches were designed to transform the student research culture on campus. The goal was to demystify science and research, highlight the relevance of science and health-related research to their communities, and emphasize the importance of diversifying the research workforce to reduce and eradicate health disparities in our society.

Off-campus outreach and recruitment included posting flyers at local community colleges, hosting information sessions at their campus, and providing online recruitment videos customized for each campus. In addition, application support workshops and office hours were offered to any interested students to assist them with their application preparation. We followed the protocols approved by the university’s Institutional Review Board for obtaining applicant and trainee (i.e., accepted student) data. More details of the outreach and recruitment are described in Kingsford et al. (2022) and Vu et al. (2023).

Applicants’ responses to “How did you hear about our program?” indicated that students heard about the NIH training programs through multiple sources (see Appendix B). The most frequently selected source was “hearing about programs through course instructors or faculty mentor,” followed by flyers/posters, other students, current trainees, and information sessions. Accepted students, particularly URMs, have also anecdotally indicated that the encouragement from their instructors and research mentors was instrumental in their decision to apply to the programs because they did not think they would be qualified for the NIH research programs.

### 2.4 Selection

The selection committee consisted of faculty program directors and faculty research mentors in the three NIH-funded programs. The application packet consisted of an application form, an academic transcript(s), a personal statement and a research interest statement (see Appendix C for prompts for these statements), and a faculty/teaching assistant recommendation form (one or two recommendation forms for LD and UD applications, respectively). The committee evaluated each student’s application holistically using an evaluation rubric (see Appendix D for the LD rubric and Appendix E for the UD selection rubric): each faculty reviewer evaluated applicants on traditional metrics (i.e., academic record, faculty recommendation, academic and career goals, and interest in health-related research) and non-traditional metrics (i.e., ability to enhance diversity of perspectives among the trainees and resiliency in the face of challenges). Student demographic information was not considered in the review. Instead, this holistic review process was implemented to ensure students from historically underrepresented groups with strong potential in a STEM research career would be identified. To increase reliability, each application was evaluated independently by two faculty members.

### 2.5 Students’ academic and demographic information

Information on campus population and applicants’ academic majors, race/ethnicity, and gender was obtained from the University records provided by the Institutional Research and Analytics (IR&A) Office. Similar information on applicants who were accepted into the program was obtained from the application forms. Majors (see Appendix A for listing) were further classified as biomedical (natural sciences or engineering), behavioral (clinical, health or social sciences) sciences, or undeclared. Biomedical disciplines included majors in two departments in College of Natural Sciences and Mathematics (Biological Sciences and Chemistry & Biochemistry) and select departments in College of Engineering (e.g., Biomedical Engineering, Chemical Engineering, Electrical Engineering, Mechanical Engineering). Behavioral disciplines include majors in select departments in College of Liberal Arts (e.g., Anthropology, Linguistics, Psychology, Sociology) and in College of Health and Human Services (e.g., Family and Consumer Sciences, Health Care Administration, Health Science, Kinesiology). Official university records were used as the primary source for identifying disciplines, but for non-matriculated applicants (transfer student applicants who were not yet in the university system) we used the data provided in the joint application. To designate historically underrepresented students in STEM and following NIH reporting requirement, applicants were categorized as either URM (i.e., African American/Black, Native American, Native Hawaiian/Pacific Islander, or Hispanic) or non-URM (Asian American, White, or Non-Hispanic multiple races). Gender was coded as male, female, or non-binary.

### 2.6 Measures of applicants’ psychosocial characteristics

Applicants’ psychosocial characteristics were measured with self-report instruments via an online survey that was sent to students upon receiving their applications. The survey was separate from the application process and was not included in the application materials that were evaluated by the selection committee. The psychosocial characteristics were collected for the purpose of program evaluation and research only and were used in the present study to determine which psychosocial characteristics are predictive of acceptance to the research training programs and thus being captured by our holistic evaluation. *Grit* was assessed with the 8-item Grit-S scale (Duckworth and Quinn, 2009) that measures the tendency to maintain passion and perseverance in working towards a long-term goal despite challenges and setbacks (e.g., I am a hard worker). The response scale ranged from 1 (does not describe me) to 5 (describes me extremely well). Four items were reverse coded. The mean score of the eight items was computed to represent the Grit composite. *Growth mindset* was assessed with the 4-item Growth Mindset scale (Midkiff et al., 2018) that measures students’ belief that success can be achieved through hard work and effort rather than fixed intelligence (e.g., No matter who you are, you can significantly change your intelligence level).

The response scale ranged from 1 (strongly disagree) to 5 (strongly agree). The mean score of four items was computed to represent the Growth Mindset composite. *Science interest* was assessed with 5 items measuring perception about enjoyment of science (e.g., I enjoy figuring out answers to scientific questions) and one item measuring problem-solving strategy (i.e., To understand science, I sometimes think about my personal experiences and relate them to topic of being analyzed). These items were adopted from the Colorado Learning Attitudes about Science Survey developed for use in biology (Semsar et al., 2011). For the current study, the word ‘biology’ was replaced with ‘science’ to measure overall science interest. Response scale ranged from 1 (strongly agree) to 5 (strongly disagree). All the 6 responses were reverse coded to represent higher numbers being indicative of higher science interest, and the mean score of the 6 items was computed. *Work value* was assessed with 3 items from the Intrinsic Awards subscale (e.g., A job where I have the chance to be curious or creative) of a measure used by Johnson and Elder (2002). Response scale ranged from 1 (not at all important) to 5 (extremely important), and the mean score of the 3 items was computed. *Self-efficacy* was assessed with the New General Self-Efficacy Scale (NGSE; Chen et al., 2001) that includes 8 items (e.g., When facing difficult tasks, I am certain that I will accomplish them) measuring how much people believe they can achieve their goals, despite difficulties. Applicants were asked to rate their feelings in relation to experiences they have had pursuing college studies on a response scale of 1 (strongly disagree) to 5 (strongly agree). The mean score of the 8 items was computed to create a composite score of Self-Efficacy.

## 3 Results

### 3.1 Comparisons of applicants and non-applicants to training programs

**FIGURE 1:**
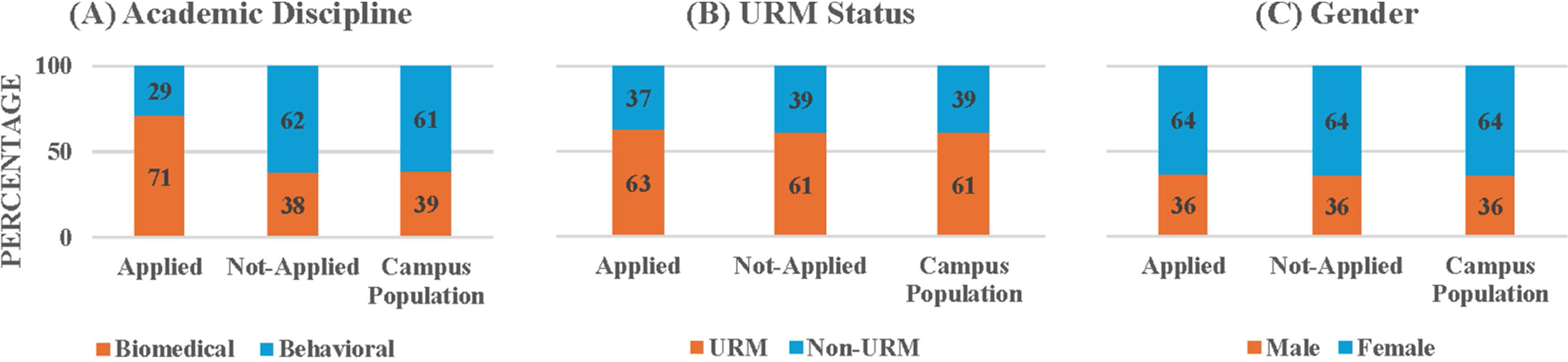
Percentages of applied and not-applied students and campus population by **(A)** academic discipline, **(B)** URM status, and **(C)** gender.

The first research question addresses the applicants’ representativeness of the diverse student population on key demographic variables associated with historical underrepresentation in STEM. Intuitively, a direct comparison of applicant demographics to those of the entire campus population would provide the most straightforward test of the applicants’ representativeness. We provide these descriptive values when presenting the results below; however, we are not able to determine whether any observed differences between the applicants and the general student population would be statistically significant as we are not aware of any statistical test that would be appropriate for such a comparison. For instance, a chi-square analysis requires that observations to be classified in one, and only one, category (i.e., assumption of independence). This independence assumption would be violated in this direct comparison because the students who applied to the program are a subset of the general student population. Thus, to address our first research question, we compared the number of students who applied to those who did not apply (i.e., their application status) across academic disciplines (biomedical vs. behavioral), URM status and gender, which allows for the Chi-Square test for Independence. Due to missing values in the demographic data provided by the IR&A office, the analytic sample size of applicants was reduced and varied across Figure 1 and Supplementary Table 1.

Figure 1A presents the percentages of students by academic discipline (biomedical vs. behavioral sciences) for the comparison between the students who applied to the NIH programs and those who did not. There were more biomedical students in the applicant pool (71.35%) compared to the campus population (38.93%). In addition, the Chi-Square test for Independence that examined whether students’ application status is associated with discipline category showed that there was a statistically significant over-representation of biomedical students in the applicant pool (71.35%) compared to the non-applicant pool (37.87%), χ^2^ (1, *N* = 28,020) = 403.03, *p* < 0.001. This result is not surprising given that two of the three training programs have long been training students in biomedical disciplines at CSULB and are thus well known among CSULB students in natural sciences.

Figure 1B presents parallel percentages for URM status (URM vs. non-URM). Numerically, there were slightly more URM students (62.71%) in the application pool compared to the campus population (60.86%). However, the Chi-Square test for Independence revealed that the application status distribution did not depend on URM vs. non-URM status, χ^2^ (1, *N* = 25,656) = 1.13, *p* = 0.29, meaning the URM percentages among applicants (62.71%) and non-applicants (60.80%) were not significantly different. This pattern was different from the general trends noted in past reports that fewer URM students applied to research programs compared to their non-URM counterparts.

Figure 1C presents parallel percentages for gender (male vs. female). Nonbinary was not included in the analysis because it was not an option available in the IR&A dataset. Numerically, there were slightly fewer females in the applicant pool (63.53%) than in the campus population (64.2%). However, the Chi-Square test for Independence showed that the application status distribution did not depend on gender, χ^2^ (1, *N* = 27,993) = 0.18, *p* = 0.68, with 63.53% females in the applicant pool compared to 64.2% females in the non-applicant pool. This suggests that the applicant pool’s gender distribution closely mirrors that of the campus population and thus male and female students were equally likely to apply to the research programs on campus, unlike the general trends previously reported.

### 3.2 Comparisons of accepted and not-accepted applicants at the lower-division level

To address the second research question about the accepted students’ representativeness of the applicant pool, we compared the distributions of accepted and not-accepted students across the academic disciplines (biomedical vs. behavioral), URM status, and gender and conducted the Chi-Square tests for Independence. These tests were performed separately for the LD and UD applicants: they were reviewed with slightly different criteria given the differential emphasis of the training programs at the LD and UD levels (early exposure for LD and intensive training for UD). We would like to note that, as was the case in section 3.1, we could not conduct a direct comparison between the accepted students and the entire applicant pool (which included the accepted students) because it would violate the independence assumption.

**FIGURE 2:**
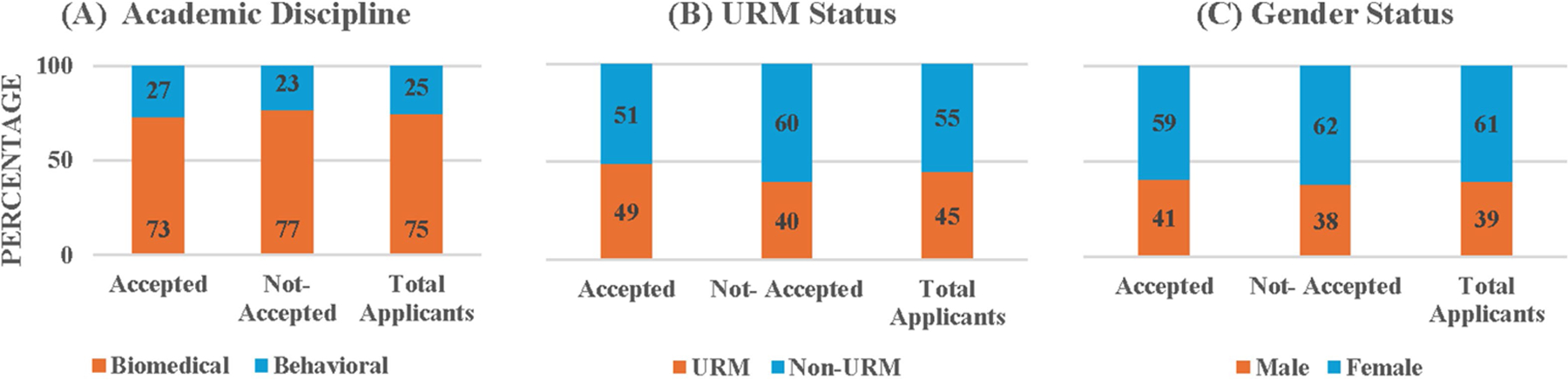
Percentages of accepted and not-accepted LD students and total applicants by **(A)** academic discipline, **(B)** URM status, and **(C)** gender.

Figure 2 (and Supplementary Table 2) presents the academic discipline and demographic data for the accepted and not-accepted LD applicants and total LD applicants, pooled across the 4 recruiting cycles of 2015–2019 during which the LD programs were implemented. It shows that biomedical students comprised 74.8% of total LD applicants whereas they comprised 73.29% of the accepted LD students. The Chi-Square Test for Independence on the association between acceptance status distribution and academic discipline showed that the distribution of acceptance status did not depend on academic discipline, χ^2^ (1, *N* = 246) = 0.43, *p* = 0.51. Next, URMs comprised 45.38% of total LD applicants whereas they comprised 48.98% of the accepted students. However, the Chi-Square Test for Independence on the association between acceptance status distribution and URM status showed that acceptance status distribution did not depend on URM status, χ^2^ (1, *N* = 249) = 1.87, *p* = 0.17. Finally, females comprised 60.56% of total LD applicants whereas they comprised 59.46% of the accepted students. Again, the Chi-Square Test for Independence showed that acceptance status distribution did not depend on gender, χ^2^ (1, *N* = 251) = 0.18, *p* = 0.67. Taken together, these results indicate that the academic discipline and demographic characteristics of students accepted into the LD program did not differ from that of the applicant pool.

**FIGURE 3:**
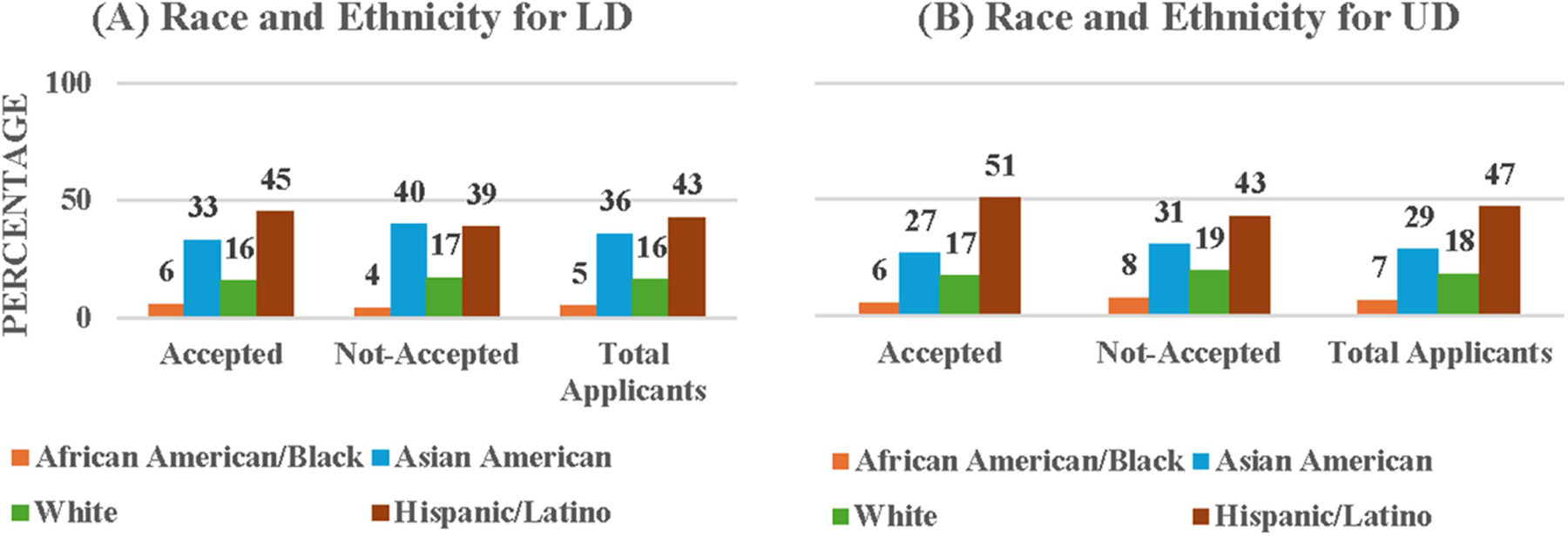
Percentages of accepted and not-accepted students by Race and Ethnicity at **(A)** LD and **(B)** UD levels.

Because our program data was more detailed than the data obtained from university records, we were able to disaggregate the race/ethnicity data for the applicants, instead of collapsing them into URM vs. non-URM categories. Figure 3 (and Supplementary Table 3A) presents the detailed race/ethnicity data for students accepted and not accepted into the LD program and the total LD applicants. The majority of LD applicants were Hispanic/Latino (42.67%) followed in the order by Asian American, White, and African American/Black. Similarly, the majority of accepted LD students were Hispanic/Latino (45.32%) followed by Asian American, White and African/Black. The Chi-Square test for Independence on the association between acceptance status and race/ethnicity showed that the acceptance status distribution did not depend on race/ethnicity, *χ*2(3, *N* = 232) = 0.18, *p* = 0.67, implying that the accepted LD students’ ethnicity and race are representative of those of the entire LD applicant pool.

### 3.3 Comparisons of accepted and not-accepted applicants at the upper-division level

**FIGURE 4:**
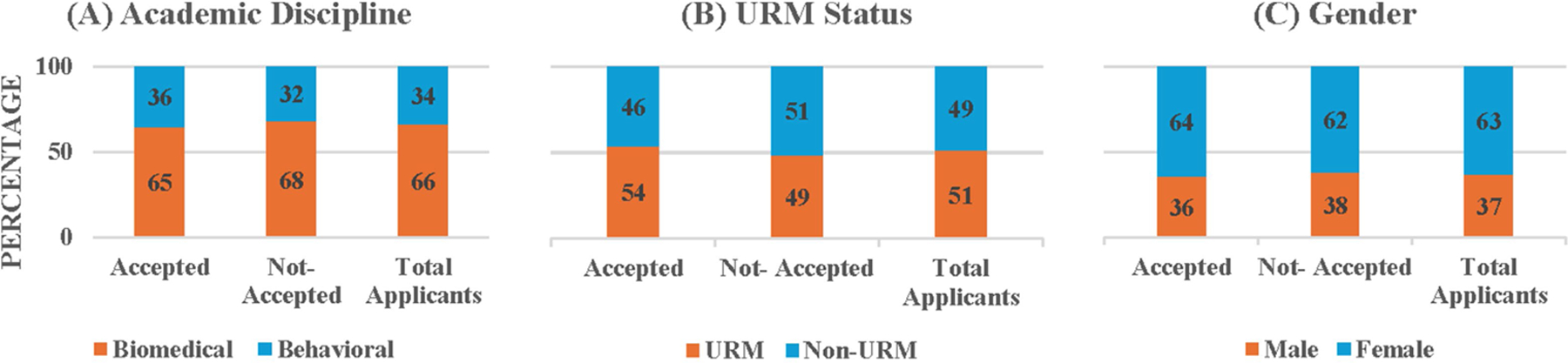
Percentages of accepted and not-accepted UD students and total applicants by **(A)** academic discipline, **(B)** URM status, and **(C)** gender.

In this section we examine the second research question for the UD program applicants. Figure 4 (and Supplementary Table 4) shows the academic discipline and demographic distributions of students who were accepted and not-accepted into the UD programs and the total UD applicants, pooled across the 8 recruiting cycles of 2015–2023. It shows that biomedical students comprised 66.09% of total UD applicants whereas they comprised 64.03% of the accepted UD students. The Chi-Square test for Independence on the association between acceptance status distribution and academic discipline showed that acceptance status distribution did not depend on discipline, χ 2(1, *N* = 805) = 1.04, *p* = 0.31. Similarly, URMs comprised 51.44% of total UD applicants whereas they comprised 53.97% of the accepted UD students. The Chi-Square test for Independence on the association between acceptance status distribution and URM status showed that acceptance status distribution did not depend on URM status, χ^2^ (1, *N* = 799) = 2.37, *p* = 0.12. In terms of gender, females comprised 62.94% of total UD applicants whereas they comprised 63.95% of the accepted UD students. Again, the Chi-Square test for Independence showed that acceptance status distribution did not depend on gender, χ^2^ (1, *N* = 804) = 0.41, *p* = 0.52. Thus, the results of these Chi-Square tests imply that the accepted UD students are representative of the entire UD applicant pool.

Figure 3 (and Supplementary Table 3B) presents the race/ethnicity distribution of students accepted and not-accepted into the UD program. The majority of applicants were Hispanic/Latino (46.93%) followed by Asian American, White, and African American/Black. Similarly, the majority of accepted students were Hispanic/Latino (50.61%) followed by Asian American, White and African/Black, suggesting a trend of more Hispanic/Latino students being selected into the training programs. However, the Chi-Square test for Independence on the association between acceptance status and race/ethnicity showed that acceptance status distribution did not depend on race/ethnicity, χ^2^ (3, *N* = 765) = 5.2, *p* = 0.16. Again, this finding implies that the accepted UD students’ ethnicity and race are representative of those of the entire UD applicant pool.

### 3.4 Predictability of acceptance for LD and UD applicants

Our final research question examines whether psychosocial characteristics of student applicants predicted acceptance to the LD versus UD research training programs. Applicants’ data on the psychological measures were not used in the selection process and were used solely for the purpose of program evaluation and research. The purpose of this research question is to determine which psychosocial characteristics known to predict various STEM outcomes, if any, are captured by our holistic selection process. Logistic regression analyses were performed, separately for LD and UD applicants, to examine whether students’ academic discipline and demographic and psychosocial characteristics can uniquely predict applicants’ acceptance to the research training programs while controlling for the rest of characteristics. The alpha level for both analyses was set at 0.05. The means, standard deviations, and correlations among psychosocial predictors are presented in Table 1. For both LD and UD applicants, all psychosocial predictors were moderately correlated with each other (0.2 ≤ *r* ≤ 0.61, *p* < 0.001 for all).

For LD applicants, only grit was a significant predictor of acceptance to the LD programs while controlling for other predictors (Table 2) such that students with one higher grit score were 2.24 times more likely to be accepted to a LD program, B = 0.81, Wald = 4.32, *p* = 0.04, OR = 2.24, 95% CI [1.05, 4.78].

For UD applicants, only science interest predicted acceptance while controlling for other predictors (Table 3) such that applicants with one higher science interest scores were 1.59 times more likely to be accepted to an UD program, B = 0.47, Wald = 4.96, *p* = 0.03, OR = 1.59, 95% CI [1.06, 2.40]. Academic discipline predictability approached statistical significance (*p* = 0.07) such that behavioral, compared to biomedical, applicants were slightly more likely to be admitted to the programs.

## 4 Discussion and conclusion

Our campus has three NIH-funded undergraduate research training programs designed to train a diverse group of undergraduate students who intend to pursue Ph.D. degrees and become researchers in health-related disciplines. We examined the demographic and psychosocial characteristics of undergraduate students who applied to these training programs and those that were accepted to the programs. We were concerned as to whether students from historically underrepresented groups were still underrepresented in the applicant pool and in the accepted pool. We used intentional strategies to appeal to URM students in our extensive outreach and recruitment efforts and used a joint application to mitigate the typical barriers URM students might face in applying to multiple research training programs. These efforts were effective as we found comparable application rates for students from historically underrepresented groups in STEM to those from our student population. As shown in Appendix B, students who applied to our program reported that they heard about the program from multiple sources. Moreover, we established a strong undergraduate research curriculum that was designed to change the research culture on campus and increase interest and access to research for our students. Our holistic selection process that could not take race, ethnicity and gender into consideration did not negatively impact the acceptance rates of the URM and female applicants into our programs. We found evidence that our selection rubrics were successful in capturing the psychosocial traits such as grit for LD applicants and science interest for UD applicants that are known to predict the successful student outcomes in STEM.

Past studies found persistent racial/ethnic disparities in pursuing research and Ph.D. programs. Black students reported to be less certain that they would pursue postbaccalaureate degree options than their White counterparts (Carter, 2001), and to be less interested in becoming a researcher in a university (Cole and Barber, 2003). Furthermore, both Black and Latinx students were less interested in research as freshmen because of lower level of SAT math scores and were less likely to be interested in a research job outside of university than Asian and White students (Cole and Barber, 2003). In addition to racial/ethnic disparity in interest in pursuing research, Herber (2018) found that German students who are the first in their family to enter college were less likely to apply for highly selective scholarships. Given the past findings, the non-underrepresentation of the URM and female students in both the overall applicant pool and the accepted student pool indicates that our extensive on-campus and off-campus outreach and recruitment strategies and the holistic selection process that incorporated psychosocial factors of science interest and grit were successful in mitigating barriers, drawing and admitting URM and female students to our research training programs.

Bastedo (2021) indicated that 95% of US universities use holistic evaluation for admissions, but what holistic evaluation meant in practice varied widely: most looked at the “whole file,” while 20% considered the “whole person,” placing emphasis on the applicant’s character, and about 30% used “whole context,” which takes into account the unique contribution of the applicant based on opportunities afforded to them by their socioeconomic and family backgrounds. Bastedo noted that the whole context approach is most ideal but not often used. Our holistic selection process aligns with the whole context approach and has objective evaluation rubric appeared to have been effective in accepting students with traits that are predictive of resilience and persistence in academia and STEM, which should capture students from historically underrepresented backgrounds. Even though applicants’ scores on the psychosocial attributes were not available to the selection committee, LD applicants with higher grit and UD applicants with higher science interest were more likely to be accepted. This result implies that criterion #6 in the selection rubric (i.e., demonstrated resilience in the face of challenge) was able to capture LD students’ grit, acknowledging the life experiences and perspectives of individuals from underrepresented groups. In fact, we found that the acceptance rate into the programs tends to be higher for URM applicants than non-URM applicants at both LD and UD levels. A potential reason that grit was not a significant predictor for UD admission is that UD students who applied to the program might already have high levels of grit. At the UD level, self-selection might have occurred where only those students high in grit applied to the program. Given that grit predicted academic success of university students (Hodge et al., 2017), our result underscores the importance of considering grit in a holistic selection process, particularly for early intervention programs at the lower division level. Science interest was a significant predictor of admission for the UD applicants and was best captured by criterion #4 in the selection rubric (i.e., strong interest in biomedical sciences, a well-articulated research question, and/or prior research). But science interest was not a significant predictor of admission for the LD applicants, which might be because a majority of the applicants may not have developed a scientific mindset or prior research experience during their first year of college. Thus, future selection rubrics might need to adopt broader language in measuring applicants’ science interest (e.g., I think about the science I experience in everyday life) as used in the science interest scale. Taken together, these findings serve as a validation of our holistic selection process and suggestions for modification for future holistic selection process.

## 5 Limitations and implications

This study has several limitations, most notably in sample size, construct validity, and missing data. First, since the study results were based on the data from one institution, sample size limitations prevent inference to the general student population across all other NIH programs at other institutions. However, CSULB represents one of the largest and most diverse institutions in the nation in terms of student population compared to other NIH program sites. Thus, the results provide important implications for the overall NIH programs. Second, there is a substantial debate on the operational definition of the grit construct such that whether two facets of grit, i.e., passion and perseverance, should be used separately or should be combined to represent grit (Credé, 2018; Guo et al., 2019). Duckworth et al. (2021) maintained that the original definition of grit with the sum of all eight items should be used for studying the construct of grit but suggested that a subset of items can be used when a researcher is particularly interested in each facet of grit. Because we intended to investigate the predictability of the overall grit on the admission to the NIH programs, we used the sum of all eight items. Duckworth et al. (2021) also proposed that the original 12 item-grit scale has better content validity. Future study should use the entire 12 items for studying the predictability of grit on a program admission. Finally, there was a sizable number of students (N = 156 for applied and N = 2,208 for not-applied groups) whose URM status was unknown from university records (e.g., they did not disclose their race or were international students). But the number of unknown/declined for URM status was less than 2 for program applicants, for which URM status can be known through self-disclosure in the application. Therefore, it does not seem that students were reluctant to answer race/ethnicity questions in their program application. The unknown URM status in the institutional data appears to reflect factors unrelated to students’ willingness to disclose their racial and ethnic background.

Despite these limitations, our findings have significant implications for future scholarships and research training programs on a local and national scale. The NIH has long funded undergraduate programs dedicated to increasing the diversity of student researchers in health-related disciplines. While it aims to aggressively tackle the persistent racial/ethnic and gender disparities in biomedical and behavioral research workforce, it has placed strict non-exclusionary policy on the selection of research trainees. Our findings are particularly important given the uncertain landscape regarding consideration of race in the U.S. college admission, including the ruling from the U.S. Supreme Court’s in 2023 that effectively overturned the use of race-conscious policies at Harvard University and the University of North Carolina at Chapel Hill. Specifically, they demonstrate that diversification of the biomedical research workforce can be achieved through the adoption of a transparent and objective holistic selection process that uses a standardized rubric that does not explicitly consider race. Proper use of this approach can help administrators avoid the historical misapplication of holistic admission processes that resulted in exclusion, rather than inclusion, of individuals from specific racial groups in the admission process decades ago (Harpalani, 2023; Wechsler, 1984). We believe that employing systematic and extensive outreach and recruitment, investing in undergraduate research curriculum, establishing a strong student-centered research culture on campus, and utilizing a transparent and objective holistic selection process that capture evidence of psychosocial characteristics predictive of resilience and persistence in STEM have together contributed to the equal representation of students from historically underrepresented groups in our campus research pipeline. Further empirical research is needed to verify our interpretation.

## Supplementary Material

supplemental materials

## Figures and Tables

**TABLE 1:** Mean (SD) and correlations among psychosocial characteristics.

	Mean (SD) for LD	Mean (SD) for UD	1	2	3	4	5
1. Grit	4.15 (0.5)	4.25 (0.50)		0.28*	0.3*	0.31*	0.59*
2. Growth Mindset	4.43 (0.6)	4.39 (0.76)	0.49*		0.24*	0.22*	0.27*
3. Science Interest	4.51 (0.43)	4.61 (0.42)	0.42*	0.35*		0.42*	0.38*
4. Work Value	4.67 (0.53)	4.74 (0.39)	0.31*	0.29*	0.2*		0.44*
5. Self-efficacy	4.41 (0.51)	4.53 (0.44)	0.61*	0.59*	0.4*	0.52*	

* *p* < 0.001.

The correlation coefficients for the lower diagonal are for LD and the upper diagonal are for UD. *N* for LD ranged from 137 to 139; *N* for UD ranged from 384 to 387.

**TABLE 2:** Demographic and psychosocial characteristics predicting LD program admission

Variables	B	S.E.	Wald	Df	*p*-value	Odds ratio	95% C.I.	
							Lower	Upper
Gender	−0.43	0.30	2.05	1	0.15	0.65	0.36	1.17
Discipline	−0.42	0.35	1.41	1	0.23	0.66	0.33	1.31
URM vs. non-URM	0.45	0.30	2.33	1	0.13	1.57	0.88	2.80
Grit	0.81	0.39	4.32	1	0.04	2.24	1.05	4.78
Growth mindset	0.14	0.25	0.33	1	0.57	1.15	0.71	1.87
Science interest	−0.32	0.39	0.69	1	0.41	0.72	0.34	1.55
Work value	0.04	0.31	0.02	1	0.90	1.04	0.56	1.93
Self-efficacy	−0.53	0.40	1.74	1	0.19	0.59	0.27	1.29
Constant	0.50	2.01	0.06	1	0.80	1.65		

**TABLE 3:** Demographic and psychosocial characteristics predicting UD program admission.

Variables	B	S.E.	Wald	Df	*p*-value	Odds ratio	95% C.I.	
							Lower	Upper
Gender	−0.07	0.17	0.18	1	0.67	0.93	0.66	1.30
Discipline	−0.32	0.18	3.30	1	0.07	0.72	0.51	1.03
URM vs. non-URM	0.26	0.16	2.53	1	0.11	1.29	0.94	1.78
Grit	−0.24	0.20	1.52	1	0.22	0.79	0.53	1.15
Growth mindset	0.01	0.12	0.01	1	0.91	1.01	0.80	1.28
Science interest	0.47	0.21	4.96	1	0.03	1.59	1.06	2.40
Work value	−0.34	0.24	2.10	1	0.15	0.71	0.45	1.13
Self-efficacy	0.17	0.23	0.50	1	0.48	1.18	0.75	1.87
Constant	0.13	1.20	0.01	1	0.91	1.14		

## Data Availability

The raw data supporting the conclusions of this article will be made available by the authors, without undue reservation.

## Footnotes

1
https://www.csulb.edu/BUILD
