# Supplementary material for: A comparison of applicant and accepted student characteristics to research training programs with implications for recruitment and selection strategy
Source: Front Educ (Lausanne). Author manuscript; Available in PMC 2025 Oct 15. (PMC12519540; doi:10.3389/feduc.2025.1474591)
Supplement: supplemental materials [file NIHMS2054960-supplement-supplemental_materials.docx]

Supplementary Tables

TABLE 1 (A) Number of applied and not-applied students and application percentage by academic discipline.

|  | Discipline | | | |  |
| --- | --- | --- | --- | --- | --- |
|  | Biomedical | | Behavioral | | Total |
|  | N | % | N | % | N |
| Applied | 630 | 71.35 | 253 | 28.65 | 883 |
| Not-Applied | 10278 | 37.87 | 16859 | 62.13 | 27137 |
| Campus population | 10908 | 38.93 | 17112 | 61.07 | 28020 |

TABLE 1 (B) Number of applied and not-applied students and application percentage by URM status.

|  | URM/Non-URM | | | |  |
| --- | --- | --- | --- | --- | --- |
|  | URM | | Non-URM | | Total |
|  | N | % | N | % | N |
| Applied | 476 | 62.71 | 283 | 37.29 | 759 |
| Not-Applied | 15138 | 60.80 | 9759 | 39.20 | 24897 |
| Campus population | 15614 | 60.86 | 10042 | 39.14 | 25656 |

Note: Although students can indicate unknown/declined for URM status, the number of cases for this category (N = 156 for applied and N = 2208 for not-applied groups) are not listed in the table as they are not part of the analysis.

TABLE 1 (C) Number of applied and not-applied students and application percentage by gender.

|  | Gender | | | |  |
| --- | --- | --- | --- | --- | --- |
|  | Male | | Female | | Total |
|  | N | % | N | % | N |
| Applied | 322 | 36.47 | 561 | 63.53 | 883 |
| Not-Applied | 9700 | 35.78 | 17410 | 64.22 | 27110 |
| Campus population | 10022 | 35.80 | 17971 | 64.20 | 27993 |

Note: Although students can indicate non-binary/declined for gender status, the number of cases for this category is small (N = 0 for applied and N = 27 for not-applied). Thus, consistent with best practices for deidentifying participants, these cases are not listed in the table.

TABLE 2 Number of accepted and not-accepted LD students and acceptance percentage by academic discipline, URM status and gender.

|  | | Academic Discipline | | | | URM/Non-URM | | | | Gender | | | | |
| --- | --- | --- | --- | --- | --- | --- | --- | --- | --- | --- | --- | --- | --- | --- |
| Acceptance Status | Overall | Biomedical Sciences | | Behavioral Sciences | | URM | | Non-URM | | Male | | | Female | |
|  | N | N | % | N | % | N | % | N | % | N | % | | N | % |
| Accepted | 146 | 107 | 73.29 | 39 | 26.71 | 72 | 48.98 | 75 | 51.02 | 60 | | 40.54 | 88 | 59.46 |
| Not- Accepted | 100 | 77 | 77.00 | 23 | 23.00 | 41 | 40.20 | 61 | 59.80 | 39 | | 37.86 | 64 | 62.14 |
| Total Applicants | 246 | 184 | 74.80 | 62 | 25.20 | 113 | 45.38 | 136 | 54.62 | 99 | | 39.44 | 152 | 60.56 |

Note: Although students can indicate unknown/declined for URM status and non-binary for gender, the number of cases for these categories was less than 2. Thus, consistent with best practices for deidentifying participants, these cases are not listed in the table.

TABLE 3 (A) Number of accepted and not-accepted LD students by race and ethnicity.

| Acceptance Status | Overall | African American/Black | | Asian American | | White | | Hispanic/Latino | |
| --- | --- | --- | --- | --- | --- | --- | --- | --- | --- |
|  | N | N | % | N | % | N | % | N | % |
| Accepted | 139 | 8 | 5.76 | 46 | 33.09 | 22 | 15.83 | 63 | 45.32 |
| Not- Accepted | 93 | 4 | 4.30 | 37 | 39.78 | 16 | 17.20 | 36 | 38.71 |
| Total  Applicant | 232 | 12 | 5.17 | 83 | 35.78 | 38 | 16.38 | 99 | 42.67 |

Note: Although students can indicate Native Hawaiian/Pacific Islander, unknown/declined, More than one race for race/ethnic status, the number of cases for these categories was less than 5. Thus, consistent with best practices for deidentifying participants, these cases are not listed in the table.

TABLE 3 (B) Number of accepted and not-accepted UD students by race and ethnicity.

| Acceptance Status | Overall | African American/Black | | Asian American | | White | | Hispanic/Latino | |
| --- | --- | --- | --- | --- | --- | --- | --- | --- | --- |
|  | N | N | % | N | % | N | % | N | % |
| Accepted | 411 | 23 | 5.60 | 110 | 26.76 | 70 | 17.03 | 208 | 50.61 |
| Not- Accepted | 354 | 27 | 7.63 | 108 | 30.51 | 68 | 19.21 | 151 | 42.66 |
| Total Applicant | 765 | 50 | 6.54 | 218 | 28.50 | 138 | 18.04 | 359 | 46.93 |

Note: Although students can indicate Native Hawaiian/Pacific Islander, unknown/declined, More than one race for race/ethnic status, the number of cases for these categories was less than 4. Thus, consistent with best practices for deidentifying participants, these cases are not listed in the table.

TABLE 4 Number of accepted and not-accepted UD students and acceptance percentage by academic discipline, URM status and gender.

|  | | Academic Discipline | | | | URM/Non-URM | | | | Gender | | | | |
| --- | --- | --- | --- | --- | --- | --- | --- | --- | --- | --- | --- | --- | --- | --- |
| Acceptance Status | Overall | Biomedical Sciences | | Behavioral Sciences | | URM | | Non-URM | | Male | | | Female | |
|  | N | N | % | N | % | N | % | N | % | N | % | | N | % |
| Accepted | 431 | 278 | 64.50 | 153 | 35.50 | 231 | 53.97 | 197 | 46.03 | 155 | | 36.05 | 275 | 63.95 |
| Not Accepted | 374 | 254 | 67.91 | 120 | 32.09 | 180 | 48.52 | 191 | 51.48 | 143 | | 38.24 | 231 | 61.76 |
| Total Applicants | 805 | 532 | 66.09 | 273 | 33.91 | 411 | 51.44 | 388 | 48.56 | 298 | | 37.06 | 506 | 62.94 |

Note: Although students can indicate unknown/declined for URM status and Non-binary for gender, the number of cases for these categories was less than 2. Thus, consistent with best practices for deidentifying participants, these cases are not listed in the table.

**Appendix A**

Admitted student majors by discipline and college

| Discipline | College | Majors |
| --- | --- | --- |
| Behavioral Sciences | College of Health and Human Services (CHHS) | Family & Consumer Sciences |
|  |  | Health Care Administration |
|  |  | Health Science |
|  |  | Kinesiology |
|  |  | Nutrition & Dietetics |
|  |  | Speech-Language Pathology |
|  | College of Liberal Arts (CLA) | Anthropology |
|  |  | Communication Studies |
|  |  | International Studies |
|  |  | Linguistics |
|  |  | Psychology |
|  |  | Sociology |
| Biomedical Sciences | College of Natural Sciences and Mathematics (CNSM) | Biological Sciences |
|  |  | Chemistry & Biochemistry |
|  | College of Engineering (COE) | Biomedical Engineering |
|  |  | Chemical Engineering |
|  |  | Electrical Engineering |
|  |  | Mechanical Engineering |

**Appendix B**

Source of recruitment advertisement and number of applicants who responded to each advertisement

| Source of advertisement | Course Instructor/Faculty Member | Info-session | Social Media | Flyers/Posters | Classroom visit | Website | Student | MARC/RISE/BUILD trainee | Other |
| --- | --- | --- | --- | --- | --- | --- | --- | --- | --- |
| N | 418 | 207 | 16 | 275 | 137 | 25 | 259 | 206 | 88 |

**Appendix C**

Prompts for personal statement and research statement in an application form

PERSONAL STATEMENT

In a separate document, please introduce yourself in 500­1000 words. Click here to download the template form. You will be asked to upload your completed document below. We are particularly interested in learning about the following:

- What are your academic and career goals?
- Tell us about any personal, educational, or professional experiences or people in your life that have shaped your academic and career goals.
- Many college students have experienced significant life challenges that impacted their education and pathway to a career. If you have overcome such challenges, what are they? How have you dealt with them?
- Lastly, tell us why you are interested in participating in a research training program? What made you decide to apply?

RESEARCH INTEREST STATEMENT

In a separate document, please explain your interest in health-related research in 500 1000 words. Click here to download the template form. You will be asked to upload your completed document below. Health-related research is represented in a number of different disciplines (e.g., engineering and the behavioral, biological, clinical, health, physical and social sciences), and can include basic, applied, and translational approaches to improving health and wellbeing. We are particularly interested in learning about the following:

- What areas of research are you curious or passionate about? Do you have a specific research question you want to answer or a problem you want to solve through research?
- What sparked your interest in these topics (e.g., a class that you took, an article you read, personal or volunteer experiences)?
- Describe the type of research that you would like to engage in over the next two years. What kinds of research knowledge and skills do you hope to gain? Try to be specific. If you are already working with a faculty mentor in research, describe the work you have done with the mentor and the research you plan to conduct over the next two years with this mentor.

**Appendix D**

**Evaluation rubric for Lower Division research training program application**

| Reviewer |  | | |
| --- | --- | --- | --- |
| Applicant Name |  | CSULB ID |  |
| Major/pre-Major |  | Cumulative GPA |  |
| College |  | Program Preference | None BUILD RISE |

**INSTRUCTIONS:** Please score each application according to the following criteria and scoring range. Scored rubrics should be saved on the shared drive or send files to Program Coordinator.

| **CRITERIA AND INDICATORS**  **SCORE RANGE: 1-4 (1-WEAK, 4 EXCELLENT) FOR CRITERIA 1-6 FOR A MAXIMUM TOTAL OF 24 POINTS.** | **SCORE** | **COMMENTS** |
| --- | --- | --- |
| **#1. ACADEMIC RECORD STRENGTH**  *Applicant excelled in college coursework. If there are overall and major GPAs, then use the higher of the two GPAs.*  4 – Applicant’s GPA > 3.6 (A average)  3 – 3.0 < Applicant’s GPA < 3.5 (B average)  2 – 2.6 < Applicant’s GPA < 2.9 (High C average)  1 – Applicant’s GPA is below 2.5 |  |  |
| **#2. ROBUSTNESS OF FACULTY REFERENCE**  *Reference form from faculty nominator indicates applicant’s academic achievement and/or potential is held in high regard.*  4 – Reference indicates very strong support or rates applicant as exceptionally high on majority of skills and traits.  3 – Reference indicates strong support or rates applicant as above average on majority of skills and traits.  2 – Reference shows some support or rates applicant’s skills and traits as mostly average.  1 – Reference shows questionable support or rates applicant’s skills and traits as mostly below average, extremely low or n/a OR inappropriate reference (e.g., reference from a friend or family member) |  |  |
| **#3. CLARITY OF ACADEMIC AND CAREER GOALS AND EVIDENCE OF PROFESSIONAL MATURITY**  *Applicant has strong interests in pursuing a career in sciences and shows signs of maturity in his/her articulation of their career goals and how he/she developed these goals.*  4 – Applicant clearly articulates in essays and/or during interview strong interests in science careers and his/her explanation reflects great maturity.  3 – Applicant articulates in essays and/or during interview solid interests in science careers and his/her explanation reflects good maturity.  2 – Applicant indicates in essays and/or during interview some interests in science careers, but the explanation does not fully support the interest.  1 – Applicant is still exploring and is relatively unsure of his/her career interests. |  |  |
| **#4. STRONG INTEREST IN BIOMEDICAL SCIENCES, A WELL-ARTICULATED RESEARCH QUESTION, AND/OR PRIOR RESEARCH EXPERIENCE**  *Applicant has articulated a clear interest in biomedical sciences, a well-reasoned science question that reflects a scientific mindset, and/or describes prior research experience cogently that demonstrates good understanding of and interest in a science career.*  4 – Applicant has defined a clear interest in biomedical sciences, an interesting science question, and/or has had experiences that significantly contributed to his/her interest in sciences.  3 – Applicant has defined a stronger interest in general or applied sciences, an appropriate science question, and/or has had experiences that contributed to his/her interest in sciences.  2 – Applicant has expressed some interest in general or applied sciences, a plausible science, clinical, or technical question, and/or has had experiences that tangentially contributed to his/her interest in science.  1 – Applicant’s science interest/question is too vague or not understandable and prior experiences do not support his/her interest in science. |  |  |
| **#5. ABILITY TO ENHANCE DIVERSITY OF PERSPECTIVES AMONG THE BUILD TRAINEES**  *Applicant’s life experiences, academic goals, and/or research interests indicate potential for diversifying perspectives in and/or approaches to biomedical research and training.*  4 – Applicant demonstrated strong potential to bring in diverse experiences and/or perspectives to the training activities.  3 – Applicant demonstrated moderate potential to bring in diverse experiences and/or perspectives to the training activities.  2 – Applicant demonstrated limited potential to bring in diverse experiences and/or perspectives to the training activities.  1 – Applicant has NOT demonstrated potential to bring in diverse experiences and/or perspectives to the training activities. |  |  |
| **#6. DEMONSTRATED RESILIENCE IN FACE OF CHALLENGES**  Applicant overcame significant barriers throughout his/her life and continued to persevere, demonstrating traits of resilience such as grit, positive self-concept, realistic self-appraisal, preference for long-term goals, and perseverance.  4 – Applicant has overcome enduring or multiple hardships.  3 – Applicant has overcome difficult circumstances.  2 – Applicant has overcome difficult circumstances.  1 – Applicant has not shown ability to overcome difficult circumstances. |  |  |
| **TOTAL SCORE** |  |  |
| **RECOMMENDATION**: ADMIT MAYBE NOT ADMIT | | |

**Appendix E**

**Evaluation rubric for Upper Division research training program application**

| Reviewer |  | | |
| --- | --- | --- | --- |
| Applicant Name |  | Student ID# |  |
| Major/pre-Major |  | Overall and major GPA |  |
| College |  | Program Preference | None BUILD MARC RISE |

**INSTRUCTIONS:** Please score each application according to the following criteria and scoring range. Scored rubrics should be saved on the shared drive or send files to Program Coordinator.

| **CRITERIA AND INDICATORS**  **SCORE RANGE: 1-4 (1-WEAK, 4 EXCELLENT) FOR CRITERIA 1-6 FOR A MAXIMUM TOTAL OF 24 POINTS.** | **SCORE** | **COMMENTS** |
| --- | --- | --- |
| **#1. Academic record strength**  *Applicant excelled in college coursework. If there are overall and major GPAs, then use the higher of the two GPAs.*  4 – Applicant’s GPA > 3.7  3 – 3.3 (B+) < Applicant’s GPA < 3.7 (A-)  2 – 2.7 (B-) < Applicant’s GPA < 3.3 (B+)  1 – Applicant’s GPA is below 2.7 |  |  |
| **#2. Robustness of letters of recommendation**  *Letters from faculty nominators indicate applicant’s academic achievement and/or potential is held in high regard.*  4 – Both letters indicate very strong support and rates applicant as outstanding (in top 5%) on majority of skills and traits  3 – At least one letter indicates very strong support and rates applicant as outstanding (in top 5%) on majority of skills and traits OR both letters indicate strong support and rate applicant as very good (in top 25%) on majority of skills and traits  2 – Letters show some support or rate applicant’s skills and traits as mostly average (upper 50%).  1 – None of the letters show support and rate applicant’s skills and traits as mostly below average (lowest 25%) or extremely low (lowest 5%) |  |  |
| **#3. Clarity of academic and career goals and evidence of professional maturity**  *Applicant has clear goals to attain a doctorate and pursue a research career and shows signs of maturity that makes it highly likely that these goals will be pursued. .*  4 – Applicant articulates in essays and/or during interview clear and convincing reasons for pursuing a doctorate and a research career that shows great potential for leadership or transformation of the biomedical field.  3 – Applicant articulates in essays and/or during interview sensible reasons for pursuing a doctorate and a research career that shows some promise for leadership or transformation of the biomedical field.  2 – Applicant indicates in essays and/or during interview a goal for pursuing some graduate education, but is not clear or sure about a doctorate degree or a research career.  1 – Applicant is still exploring and is relatively unsure of pursuing a graduate education or a research career. |  |  |
| **#4. Strong interest in scientific research, a well-articulated research question, and/or prior research experience**  *Applicant has articulated a clear research interest or question that reflects a scientific mindset, and/or describes prior research experience cogently that demonstrates good understanding of and interest in the scientific research process.*  4 – Applicant has defined a clear and specific research interest/question, and/or has had extensive scientific research experience (a semester or more).  3 – Applicant has defined a general research interest/question, and/or has had some scientific research experience (less than a semester).  2 – Applicant has defined a vague research topic, and has not had any research experience.  1 – Applicant’s research interest/question is too vague or not understandable. |  |  |
| **#5. Ability to enhance diversity of perspectives among the BUILD trainees**  *Applicant’s life experiences, academic goals, and/or research interests indicate potential for diversifying perspectives in and/or approaches to biomedical research and training.*  4 – Applicant demonstrated strong potential to bring in diverse experiences and/or perspectives to the training activities.  3 – Applicant demonstrated moderate potential to bring in diverse experiences and/or perspectives to the training activities.  2 – Applicant demonstrated limited potential to bring in diverse experiences and/or perspectives to the training activities.  1 – Applicant has NOT demonstrated potential to bring in diverse experiences and/or perspectives to the training activities. |  |  |
| **#6. Demonstrated resilience in face of challenges, while still making academic progress**  Applicant overcame significant barriers throughout his/her life and continued to make solid accomplishments, demonstrating traits of resilience such as grit, positive self-concept, realistic self-appraisal, preference for long-term goals, and perseverance.  4 – Applicant has overcome enduring or multiple hardships and has made solid academic progress.  3 – Applicant has overcome difficult circumstances and has made decent academic progress.  2 – Applicant has overcome difficult circumstances but has struggled in making academic progress.  1 – Applicant did NOT address or demonstrate an ability to overcome difficult circumstances. |  |  |
| **TOTAL SCORE** |  |  |
| **Recommendation for Bonus Points (+2). Extraordinary and/or unique factors not considered in criteria #1-6**  Essays and/or interview reveal extraordinary or unique circumstances that highlight applicant’s potential to succeed that was not captured adequately with above criteria. The bonus points are assigned AFTER all applications are reviewed to no more than 15% of total applicants. |  |  |
| **RECOMMENDATION: ADMIT MAYBE NOT ADMIT** | | |
